# Consultation trends in patients admitted to a Psychiatric Emergency Service before and during COVID-19 pandemic

**DOI:** 10.1192/j.eurpsy.2023.1236

**Published:** 2023-07-19

**Authors:** M. Gomez Ramiro, A. Gimenez, G. Fico, M. Sague Vilavella, M. Valenti, M. Vazquez

**Affiliations:** 1 Psychiatry, hospital alvaro cunqueiro, Vigo; 2 Psychiatry, hospital clinic, Barcelona, Spain

## Abstract

**Introduction:**

The COVID-19 outbreak had significant implications worldwide, including mental health. Consultations in the Emergency Service of the Hospital Clínic of Barcelona varied in terms of reasons for consultations, psychopathology, and other aspects, before and during the pandemic.

**Objectives:**

This study aims to assess differences in the profile of patients admitted within the last three years to the Psychiatric Emergency Service of a third-level hospital, in order to analyze variations in the number of patients visited, diagnoses and admissions throughout the different seasons.

**Methods:**

All adults admitted from 2019 to 2021 to the Psychiatric Emergency Service of Hospital Clínic of Barcelona, Spain, were retrospectively included for analysis and divided into three groups depending on the year they attended the Emergency Service. SPSS v25.0 and R statistics were used in order to compare differences between groups.

**Results:**

A total of 13677 adult individuals who attended the psychiatric emergency service of Hospital Clínic of Barcelona between 2019 and 2021 were included in the analysis. 4814 patients were visited in 2019, 4007 in 2020 and 4856 in 2021. The majority of patients were male (50.1%), with a mean age of 40.47 years (SD 15.83). In terms of acute admission rates, 24.6% of the total sample were hospitalized in an acute psychiatric unit, whereas in the spring of 2020, 34.3% of patients attending the Emergency Service were hospitalized. This revealed significant differences when compared with spring of 2019 and 2021 and with the rest of seasons (p<0.05). With regard to suicide attempts and intentional poisonings, significant differences were only observed between winter of 2019, with the lowest rate, and autumn of 2020, with the highest proportion. In spring of 2019, the lowest rate of patients attending with suicidal ideation was observed, which showed significant differences compared to winter of 2020, spring of 2021, summer of 2021 and autumn of 2021 (p<0.05). Also, statistically significant differences between winter of 2019 and summer of 2021 and also between summer of 2019 and summer of 2021 were observed, with the highest rate in the last one. No significant differences were observed in rates of patients with severe mental disorders visited.

**Conclusions:**

The COVID-19 pandemic and the situation of lockdown lead to an overall reduction in the overall consultations to the Emergency Service, being this reduction non-significant in patients with severe mental disorders, such as psychotic disorders. In addition, our study shows a decrease in consultations with suicidal ideation in summer of 2019 and a significant increase in summer of 2021. In contrast, an increased tendency in suicide attempts was not observed.

**Disclosure of Interest:**

None Declared

